# Implant removal of osteosynthesis: the Dutch practice. Results of a survey

**DOI:** 10.1186/1752-2897-6-6

**Published:** 2012-08-03

**Authors:** Dagmar Vos, Beate Hanson, Michiel Verhofstad

**Affiliations:** 1 Department of Surgery, Amphia Hospital Breda, PO Box 90158, Breda, 4800 RK, Netherlands; 2 AO Clinical Investigation and Documentation, Dübendorf, Switzerland; 3 Department of Surgery, St. Elisabeth Hospital, Tilburg, Netherlands

**Keywords:** Osteosynthesis, Implant removal, Survey, Complaints, Fracture healing

## Abstract

**Background:**

The aim of this survey study was to evaluate the current opinion and practice of trauma and orthopaedic surgeons in the Netherlands in the removal of implants after fracture healing.

**Methods:**

A web-based questionnaire consisting of 44 items was sent to all active members of the Dutch Trauma Society and Dutch Orthopaedic Trauma Society to determine their habits and opinions about implant removal.

**Results:**

Though implant removal is not routinely done in the Netherlands, 89% of the Dutch surgeons agreed that implant removal is a good option in case of pain or functional deficits. Also infection of the implant or bone is one of the main reasons for removing the implant (> 90%), while making money was a motivation for only 1% of the respondents. In case of younger patients (< 40 years of age) only 34% of the surgeons agreed that metal implants should always be removed in this category. Orthopaedic surgeons are more conservative and differ in their opinion about this subject compared to general trauma surgeons (*p* = 0.002). Though the far majority removes elastic nails in children (95%).

Most of the participants (56%) did not agree that leaving implants in is associated with an increased risk of fractures, infections, allergy or malignancy. Yet in case of the risk of fractures, residents all agreed to this statement (100%) whereas staff specialists disagreed for 71% (*p* < 0.001). According to 62% of the surgeons titanium plates are more difficult to remove than stainless steel, but 47% did not consider them safer to leave in situ compared to stainless steel. The most mentioned postoperative complications were wound infection (37%), unpleasant scarring (24%) and postoperative hemorraghe (19%).

**Conclusion:**

This survey indicates that there is no general opinion about implant removal after fracture healing with a lack of policy guidelines in the Netherlands. In case of symptomatic patients a majority of the surgeons removes the implant, but this is not standard practice for every surgeon.

## Background

Indications for implant removal are not well defined in clinical protocols and there is ongoing discussion concerning this issue worldwide [1,2]. Yet implant removal is a procedure that is often done, despite the frequency differs among countries. In the Netherlands alone, about 18 000 operations for implant removal after fracture healing are performed each year [3]. The *Arbeitsgemeinschaft für Osteosynthesefragen* (AO), founded in 1958, advised removing all materials as a standard, especially in the lower extremity [4]. This statement has been done at the time when most of the implants were made of stainless steel. The discussion has intensified since the evolution of titanium implants. Titanium components have become more popular because the alloy is considered to have better biomechanical properties and to be safe to leave in situ [5,6]. Moreover, removing titanium implants can be very difficult due to bony overgrowth or stripping of the screw head in angular stable constructs [7-9]. These factors provide arguments for the antagonists of implant removal. In literature only three prospective single centre cohort study’s handling about the outcome of implant removal can be found [10-12] and there is an absence of randomized prospective trials. In the absence of guidelines of conduct, many surgeons consequently still decide at their own discretion to remove implants or leave them in situ.

In the Netherlands, fracture surgery and subsequent implant removal is mainly performed by trauma surgeons (approximately 70%) and orthopaedic surgeons (approximately 30%). The purpose of this study was to evaluate the current attitudes and practice of both types of surgeons toward implant removal, particularly in light of the current number of removals performed in the Netherlands.

## Methods

After permission was obtained from the boards of both the Dutch Trauma Society and Dutch Orthopaedic Trauma Society, all 540 active members were invited by email to fill out a highly secured web-based questionnaire (SurveyMonkey™) comprising 44 items to determine habits, beliefs, assumptions and opinions about implant removal. In order to optimize the response rate, the email request was sent twice. Finally, a paper version was distributed during a national trauma congress among the trauma and orthopaedic surgeons who did not fill in the web-based questionnaire. There was no financial or non-financial incentive for participants to complete the survey.

The questionnaire was similar to the 41-item survey developed by Hanson *et al.*[2] and extended with 3 questions regarding payment issues. The english version of the 44-item questionnaire is presented in Appendix  1. The questionnaire has finally been revised by an epidemiologist and reviewed by all authors until consensus was achieved. Overall, there were 6 general demographic questions, 12 questions on general opinion and payment issues, 15 specific implant removal policy questions, and 11 questions about personal ideas and habits. General beliefs were measured using 6-point Likert scales with the following answer options: ‘I strongly agree’, ‘I agree’, ‘I sometimes agree’, ‘I don’t know’, ‘I disagree’, and ‘I strongly disagree’. For specific questions concerning implant removal policy, the ratings were based on a 5-point Likert scale ranging from ‘never’, ‘sometimes’, ‘often’, ‘always’ to ‘no opinion’. All other questions could be answered with ‘yes’ or ‘no’.

All data were analysed using standard descriptive statistics to allow comparison with the original questionnaire [2]. Results are presented as proportions, means ± standard deviations or medians with their ranges of distribution and 95% confidence intervals (CI).

Multivariable logistic models were fitted to the data using Firth’s penalised likelihood estimation [13] to investigate how factors were associated with the likelihood of agreeing to each of the 12 general opinion and payment issue statements. The factors considered to be at influence on this agreement were pre-specified allowing comparison with the original questionnaire [2]: age, gender (female vs. male), background (orthopaedic surgeon vs. trauma surgeon), position (resident and fellow vs. staff specialist), affiliation (non-academic teaching hospital vs. others) and employment status (contract employment vs. self employment). In order to perform the logistic regression, outcomes were dichotomised into the categories of agreement (i.e. when a surgeon agreed with a statement and provided one of the following answers: “sometimes agree”, “agree”, or “fully agree”) and disagreement (i.e. when a surgeon did not agree with a statement and provided one of the following answers: “disagree” or “fully disagree”). Assuming that all surgeons who did not provide any opinion would have disagreed, a conservative sensitivity analysis was done. The results did not change the main analysis; therefore this group was not included in the regression analysis.

## Results

From 540 potential participants, 250 questionnaires were completed (response rate 46%). The demographic profile of the respondents is presented in Table 1. The majority of respondents were trauma surgeons (180/250; 72%) and 17% (44/250) of the total group were residents. The average age was 46 years. The respondents worked at varying affiliations with approximately half based in non-academic teaching hospitals. Also, half of the respondents worked in contract employment with the other half employed in their own practice.

**Table 1:** **Demographic profile of respondents ( *n* ** **= 250)**

	**n**	**[%]**
**Age** (years; mean ± s.d.)	46 ± 10	
**Gender**		
Male	234	94
Female	16	6
**Professional background**		
Trauma surgery	180	72
Orthopaedic surgery	69	28
Plastic surgery	1	0
**Affiliation**		
University hospital	64	26
Non-academic teaching hospital	132	53
Non-academic non-teaching hospital	49	20
Private clinic	5	2
**Current position**		
Staff specialist	199	80
*Trauma surgery*	*140*	
*Orthopaedic surgery*	*58*	
*Plastic surgery*	*1*	
Trauma fellow	7	3
Resident	44	17
*Trauma surgery*	*31*	
*Orthopaedic surgery*	*11*	
*Junior*	*2*	
**Employment status**		
Contract employment	121	48
Self employment	129	52

**Figure 1:**
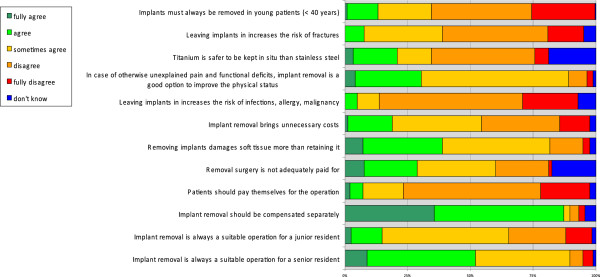
How would you rate the following statements.

In response to the questionnaire statement regarding the examination of younger patients (< 40 years of age), only 34% (95%CI: 29;41%) of the respondents agreed with the question that metal implants should always be removed for this patient category (Figure 1). When considering the type of surgeon (i.e. orthopaedic vs. trauma surgeon) and its effect on agreeing with statement 1, orthopaedic surgeons were less likely to agree that implants should always be removed in the < 40 year patient group compared to general surgeons (*p* = 0.002) (Table 2).

**Table 2:** Multivariable logistic regression analysis of the 12 general opinion and payment issues questionnaire statements

**Statement #**^**a**^	** *n* **	**Factor**					
		** *Age* **	** *Gender* **	** *Background* **	** *Position* **	** *Affiliation* **	** *Employment* **
**1**	243	1.00 (0.96;1.04)	1.27 (0.41;3.94)	** *0.32 (0.16;0.66)* **^**c**^	2.18 (0.84;5.66)	0.91 (0.49;1.70)	0.87 (0.41;1.84)
**2**^**b**^	232	1.03 (0.99;1.08)	2.60 (0.53;12.71)	1.35 (0.65;2.82)	** *439.82* **^**c,d**^** *(23.6;8211.3)* **	1.53 (0.72;3.25)	0.99 (0.44;2.20)
**3**	198	1.01 (0.97;1.05)	1.86 (0.53;6.48)	0.76 (0.40;1.45)	2.38 (0.85;6.63)	1.27 (0.68;2.40)	0.92 (0.43;1.97)
**4**	241	0.99 (0.93;1.05)	1.81 (0.22;15.18)	0.65 (0.26;1.65)	0.92 (0.20;4.14)	0.50 (0.19;1.35)	0.54 (0.17;1.65)
**5**	226	1.02 (0.97;1.08)	2.56 (0.74;8.89)	0.40 (0.14;1.12)	1.85 (0.52;6.57)	1.07 (0.44;2.56)	1.98 (0.68;5.73)
**6**	238	1.00 (0.96;1.03)	1.65 (0.54;5.02)	1.75 (0.95;3.20)	0.88 (0.35;2.20)	0.62 (0.35;1.12)	0.57 (0.29;1.13)
**7**	238	1.01 (0.96;1.06)	0.93 (0.23;3.67)	0.81 (0.37;1.78)	0.51 (0.15;1.80)	0.52 (0.23;1.14)	1.27 (0.47;3.47)
**8**^**b**^	201	1.01 (0.97;1.05)	10.31 (0.59;178.94)	0.90 (0.45;1.81)	1.24 (0.39;3.96)	1.60 (0.81;3.16)	1.34 (0.60;2.98)
**9**	238	0.96 (0.92;1.00)	1.78 (0.58;5.46)	0.89 (0.44;1.81)	0.59 (0.20;1.67)	0.93 (0.47;1.82)	0.79 (0.35;1.78)
**10**	233	0.98 (0.91;1.06)	0.93 (0.10;8.41)	1.12 (0.33;3.80)	1.84 (0.28;12.20)	0.35 (0.10;1.28)	0.30 (0.08;1.17)
**11**	240	1.02 (0.98;1.06)	2.79 (0.74;10.57)	0.53 (0.29;0.97)	0.92 (0.35;2.42)	0.84 (0.46;1.54)	1.07 (0.53;2.18)
**12**	241	0.97 (0.92;1.03)	1.14 (0.13;9.89)	1.01 (0.37;2.77)	1.06 (0.19;5.81)	1.25 (0.46;3.40)	0.89 (0.29;2.70)

The tabulated numbers are odds ratios with their 95 % confidence intervals (in parentheses).

^a^ See Appendix 1 for details of the questionnaire statements.

^b^ Logistic regression estimation based on Firth’s penalised likelihood.

^c^ Significant odds ratio at *p* = 0.05.

^d^ Though the effect is huge because 100 % of the residents agreed to this statement, the ‘position’ perfectly predicts agreement to statement 2.

In response to the general opinion statements 2 and 5, most of the participants (56%; 95%CI: 50;63% vs. 79%; 95%CI: 74;84%) did not agree that leaving implants in is associated with an increased risk of fractures, infections, allergy or malignancy (Figure 1). Yet residents and fellows agreed for 100% with statement 2 (i.e. leaving implants in increases the risk of fractures) whereas 71% of the staff specialists disagreed with this statement (*p* < 0.001) (Table 2).

In general, titanium was not considered safer when left in compared to their stainless steel implants (47%; 95% CI: 40;53%), and the removal of implants was seen as a good option in cases of unexplained pain or functional deficits (89%; 95% CI: 85;93%). Nevertheless, the majority of respondents (82%; 95%CI: 76;86%) still agreed that removing an implant damages soft tissue more than retaining it (Figure 1).

In consideration of the costs associated with implant removal, 54% (95%CI: 48;61%) of the participants agreed that implant removal involves unnecessary costs, but only 23% (95%CI: 18;29%) believes that patients should pay for this procedure by themselves (Figure 1). The majority (90%; 95%CI: 85;93%) agreed that implant removal should be separately paid for and not included in the payment for primary fracture treatment.

Sixty-five percent of the survey population (95%CI: 59;71%) agreed that implant removal surgeries are suitable for junior residents, even though 90% (95%CI: 85;93%) acknowledged that such an operation is more suitable for a senior resident (Figure 1).

The majority of the surgeons (77%; 95%CI: 71;82%) agreed that specific implants such as distal radius plates and femoral intramedullary nails should be left in situ (Table 3). In addition, cerclage wires for olecranon (76%; 95%CI: 71 - 82%) and patella fixation (74%; 95%CI: 67 – 80%) were mentioned to be mostly removed.

**Table 3:** **Surgeons’ opinions on the removal of specific implants**^**a**^

**Location**	**Implant type**	**Possible answer [%]**				
		** *Always* **	** *Often* **	** *Sometimes* **	** *Never* **	** *No opinion* **
** *Upper extremity* **						
*Olecranon*	*Tension band*	21	56	23	0	0
*Clavicle*	*Shaft plate*	6	40	47	6	2
*Radius*	*Shaft plate*	0	12	67	18	2
*Radius*	*Distal plate*	0	12	73	13	2
*Humerus*	*Distal plate*	0	7	75	17	1
*Humerus*	*IM nail*^*b*^	1	4	72	22	2
*Humerus*	*Proximal plate*	0	2	80	17	1
*Humerus*	*Shaft plate*	0	1	59	40	0
** *Lower extremity* **						
*Patella*	*Tension band*	22	52	24	1	1
*Tibia*	*Plate*	3	23	66	5	2
*Fibula*	*Plate*	1	24	68	5	2
*Tibia*	*IM nail*	1	20	71	5	3
*Femur*	*Plate (incl. SHS*^*c*^*)*	1	8	75	14	2
*Femur*	*IM nail*^*b*^	0	12	73	13	2
** *In children* **	*Elastic nail*	72	23	4	0	1

The tabulated numbers represent percentages of the total number of respondents (*n* = 250).

^a^ The question answered by the respondents was: “*In your opinion, do you think the following implants should be removed?*”.

^b^ IM = intramedullary.

^c^SHS = sliding hip screw.

Also in children, 72% (95%CI: 66;77%) mentioned always to remove elastic nails.

Individual reasons for implant removal varied among the respondents and are summarized in Table 4. Infection of the implant or bone and specific complaints made by the patient were the main reasons for implant removal (> 90%), while performing the same service to make money was a motivation for only 1% (95%CI: 0;3%) of the respondents. Nearly all of the respondents (249/250) agreed that implant removal could improve pain and pressure on skin or soft tissue. Over half of the survey population also agreed that implant removal would improve functional deficits (76%) and reduce swelling (59%) and paresthesia (47%).

**Table 4:** Various reasons for implant removal and its consequences based on surgeons’ opinions

	**Answer**^a^	
	** *Yes* [%]**	** *No* [%]**
**What are reasons for you to remove metal implants?**		
Money maker	1	99^b^
No specific reason	3	97
Bad experience leaving implant in	7	93
That's how I learned it	10	90
To avoid future surgical problems	30	70
To avoid future complications	42	58
Implant breakage	63	37
On patient's request	68	32
In case of children	84	16
In case of specific patient complaints	92	8
Infection	94	6
**Which patient complaints can improve by removing metal implants?**		
Pressure of the skin or soft tissue	97	3
Pain	94	6
Limited range of motion	76	24
Swelling	59	41
Paresthesia	47	53
Problems with daily living	20	80

The numbers represent percentages of the total number of respondents (*n* = 250).

^a^ Percentages are derived from a total of 163 respondents who answered ‘yes’ or ‘no’ to this specific item.

^b^ 87 surgeons employed by contract did not provide an answer for the reason “money maker” because it was not applicable to their working situation.

**Figure 2:**
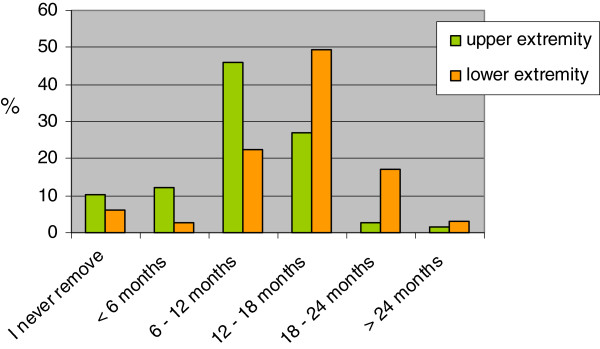
How many months after fracture consolidation do you remove the implant.

Figure 2 shows that approximately half of all respondents (46%; 95%CI: 40;53%) removes implants after fracture consolidation in the upper extremity 6 to 12 months after application, whereas this is done after 12 to 18 months in the lower extremity (49%; 95%CI: 43;56%).

The survey of peri- and postoperative problems encountered is presented in Table 5. The far most frequently experienced problem during the removal surgery is bony overgrowth (85%). Surgery that took longer than planned, stripping of screw heads and cold welding were documented as further operative obstacles for over half of the surgeons (57 – 66%). Sixty-two percent (95%CI: 55-67%) of the respondening surgeons agreed that titanium plates are more difficult to remove than stainless steel implants. The most commonly estimated postoperative complications were wound infection (37%; 95%CI: 31; 43%), unpleasant scarring (24%; 95%CI: 19;30%) and postoperative hemorraghe (19%; 95%CI: 14;24%). Notably, 13% (95% CI: 9;18%) reported that they never observe complications after removal.

**Table 5:** Per- and postoperative problems encountered from implant removal operations

	**[%]**^**a**^
** *Peroperatively* **	
Nerve damage	2
Bleeding	13
Titanium nail is more difficult to remove	19
Implant breaks during removal	20
Incorrect instruments present	34
Unplanned fluoroscopy	37
Titanium plate is more difficult to remove	55
Cold welding	57
Stripping screw head	62
Implant difficult to find	62
Enlargment of original incision	63
Operation time longer than planned	66
Implant bone overgrowth	85
No problems observed	4
** *Postoperatively* **	
Nerve damage	1
Persisting complaints	2
Refracture	3
Bleeding	19
Unpleasant scar	24
Wound infection	37
Others	1
No complications observed	13

^a^ The total number of respondents is 250.

## Discussion

Our study shows that the current practice and attitude toward implant removal, amongst trauma and orthopaedic surgeons in the Netherlands, varies in our relatively small single European country. Similar variations have also been described in few other European countries. In Finland, implant removal is more or less routinely done, resulting in a removal rate of approximately 80% [14], whereas in Norway removal is merely done in patient with complaints (removal rate around 50%) [15]. For Great Britain, the total percentage of removals is quite low at about 20% [16]. These percentages suggest that the daily practice of implant removal is determined by expert-based or cultural factors instead of evidence-based knowledge about the true functional outcomes of this surgical procedure.

Practice is mostly based on personal beliefs of benefit for the patient. In our survey there seems to be consensus that specific hardware related complaints like pain and pressure on the skin or soft tissue irritation can be improved by implant removal, as these are the most frequently mentioned reasons to take an implant out (e.g. tension bands on olecranon or patella and plates on clavicle or tibia). Dodenhoff *et al.*[17] investigated the relief of pain after the removal of femoral nails. Although there was some uncertainty as to whether pain stemmed from the femoral nail or was due to heterotrophic ossification, the majority of patients were relieved. Removal of tibial nails in patients with anterior knee pain relieves pain in 45-88% [18-20]. However, in one of these studies 3 out of 18 patients who were asymptomatic before implant removal, developed long term complaints afterwards [19].

Implant removal in young patients because of prophylactic reasons, especially in the lower extremity, is frequently advocated in view of potential future surgeries, such as joint replacement or operative treatment of new fractures. These operations will be more difficult to perform if metal implants remain in situ. Another reason for implant removal in young patients, according to some authors, is the possible risk of a refracture due to the implant itself, for example in the forearm, whereas removal after many years can be more difficult due to bone overgrowth [11,21,22]. One can suppose that removing the material at an earlier time point is therefore of benefit to avoid such situations. Currently, there is no evidence on this specific issue. Although a majority of the Dutch surgeons believes that implants should be routinely removed in children, for adult patients - including those under the age of 40 years - routine removal is not advocated. Particularly the Dutch orthopaedic surgeons appear more reticent.

One of the main outcomes of implant removal after fracture healing includes implant removal related complications. The estimated risks for these adverse events vary in the literature from 1% for postoperative bleeding, 0 to 14% for wound infection, 1 to 29% for nerve damage, 1 to 30% for a refracture up to 9% for obtaining an unpleasant scar [10,23-30]. Unfortunately, most of the data originate from older publications and there are very few recent studies reporting on these specific complications. The most commonly mentioned postoperative complications in our survey were wound infection (37%), unpleasant scarring (24%) and postoperative hemorraghe (19%), though these numbers estimated.

Another reason for the wide variance in the (international) practice of implant removal might be the associated costs. It is a fact that each operation has its costs, and implies a recovery period and temporary employment loss with social consequences [1,14,15]. In the absence of evidence based guidelines, the availability of workers compensation might affect patient’s opinion to implant removal. On the other hand, the compensation for direct costs for the doctor and hospital and the availability of requested hospital resources might also influence the removal rate. Some surgeons from our collective find that the procedure is not adequately paid for. This could be a reason not to remove metal implants unless a patient suffers a lot of complaints, though these financial incentives did not appeared to play a significant role in the decision making.

Comparing our survey to that of Hanson *et al.*[2], the demographic profile of the respondents differed slightly. The interviewees in Hanson’s study were more frequently residents (34%) as can be expected in an AO-course, and came from 65 different countries all over the world, mostly with a general orthopaedic background. Our study population originated from a single, westeuropean country and contained a lot of staff surgeons dedicated to and experienced in fracture surgery. The large majority (92%) of the Dutch trauma and orthopaedic surgeons decide to remove implants in symptomatic patients, whereas in the surgeon collective of Hanson *et al.* patients’ requests and complaints were of less importance in the decision-making process (69%). Remarkably, surgeons in that study were less enthusiastic about the beneficial effect of implant removal in ‘symptomatic’ patients. In our study, orthopaedic surgeons were less likely to agree that implants should always be removed under the age of 40 years compared to trauma surgeons with a general surgical background. A similar majority of all surgeons in both studies did not favour routine implant removal in asymptomatic patients, though more surgeons in our study felt that removing material was of greater risk to soft tissues than leaving it in situ (84% versus 50% in Hanson’s study). Overall, the variety of views reported is indicative for the large differences in opinion and attitude about implant removal between surgeons from different backgrounds and countries. Despite the demographic differences between both studies, the results are quite comparable.

Our study only describes personal opinions and habits regarding implant removal of the practicing surgeons in the Netherlands. Although effort was made to make the survey as complete as possible, it is generally known that questionnaire surveys are prone to multiple sources of bias [31].

## Conclusions

Independent of its limitations, this survey study indicates that there is no general opinion and attitude about implant removal after fracture healing within a small country as the Netherlands. As in other countries, many different habits and ideas appear to exist about this subject and the wide variety of answers given in our survey favours the necessity to develop guidelines. Our study group is now performing a multicentre cohort study to provide answers to a lot of questions concerning functional outcome of removal of specific implants. Such data are necessary for development of general policy directives on implant removal. As long as guidelines are lacking, surgeons seem to do what they think is best and individual patient complaints appear to play an important role in the decision making.

## Competing interests

The author(s) declare that they have no competing interests.

## Authors' contributions

DV has made substantial contributions to conception, study design, data analysis and interpretation, and writing the manuscript. BH has revised the study design and preliminary manuscript critically. MV has made substantial contributions to conception and study design, checked data interpretation and revised the manuscript. All authors have given final approval of the version to be published.

## Supplementary Material

Additional file 1 **Appendix 1.** The 44-item implant removal questionnaire sent to the Dutch participants. Click here for file
