# Supplementary material for: Implant removal of osteosynthesis: the Dutch practice. Results of a survey
Source: J Trauma Manag Outcomes. 2012 Aug 3;6:6. doi: 10.1186/1752-2897-6-6 (PMC3485133; doi:10.1186/1752-2897-6-6)
Supplement: Additional file 1 — Appendix 1. The 44-item implant removal questionnaire sent to the Dutch participants. [file 1752-2897-6-6-S1.doc]

**Appendix 1** The 44-item implant removal questionnaire sent to the Dutch participants (English version)

**General demographic questions (6 items)**

| **1. Age** |
| --- |
| **2. Gender** |
| Male |
| Female |
| **3. Professional background** |
| Trauma surgery |
| Orthopaedic surgery |
| Plastic surgery |
| **4. Affiliation** |
| University hospital |
| Non-academic teaching hospital |
| Non-academic non-teaching hospital |
| Private clinic |
| **5. Current position** |
| Staff specialist |
| *Trauma surgery* |
| *Orthopaedic surgery* |
| *Plastic surgery* |
| Trauma fellow |
| Resident |
| *Trauma surgery* |
| *Orthopaedic surgery* |
| *Junior* |
| **6. Employment status** |
| Contract employment |
| Self employment |

**Statements on general opinion and payment issues (12 items)**

***Answer options: ‘I strongly agree’, ‘I agree’, ‘I sometimes agree’, ‘I don’t know’, ‘I disagree’, ‘I strongly disagree’***

| 1. Implants must always be removed in young patients (< 40 years) |
| --- |
| 2. Leaving implants in increases the risk of fractures |
| 3. Titanium is safer to be kept in situ than stainless steel |
| 4. In case of otherwise unexplained pain and functional deficits, implant removal is a good option to improve the physical status |
| 5. Leaving implants in increases the risk of infections, allergy, malignancy |
| 6. Implant removal brings unnecessary costs |
| 7. Removing implants damages soft tissue more than retaining it |
| 8. Removal surgery is not adequately paid for |
| 9. Patients should pay themselves for the operation |
| 10. Implant removal should be compensated separately |
| 11. Implant removal is always a suitable operation for a junior resident |
| 12. Implant removal is always a suitable operation for a senior resident |

**Specific implant removal policy questions (15 items)**

***Answer options: ‘never, ‘sometimes’, ‘often’, ‘always’, ‘no opinion’***

| **In your opinion, do you think the following implants should be removed?** |
| --- |
| 1. Elastic nails in children |
| 2. Plate fibula |
| 3. Intramedullary (IM) nail tibia |
| 4. Plate tibia |
| 5. Tension band patella |
| 6. IM nail femur |
| 7. Plate femur (incl. sliding hip screw [SHS]) |
| 8. Plate distal radius |
| 9. Plate radius |
| 10. Tension band olecranon |
| 11. IM nail humerus |
| 12. Plate distal humerus |
| 13. Plate humerus shaft |
| 14. Plate proximal humerus |
| 15. Plate clavicle |

**Personal ideas and habits (11 items)**

*Answer options: ‘yes’, ‘no’*

| **1. What are normally reasons for you to remove metal implants?** |
| --- |
| Money maker |
| No specific reason |
| Bad experience leaving implant in |
| That's how I learned it |
| To avoid future surgical problems |
| To avoid future complications |
| Implant breakage |
| On patient's request |
| In case of children |
| In case of specific patient complaints |
| Infection |

| **2. Which patient complaints can improve by removing the implant?** |
| --- |
| Pressure of the skin or soft tissue |
| Pain |
| Limited range of motion (ROM) |
| Swelling |
| Paresthesia |
| Problems with daily living |

| **3. How many months after consolidation of the fracture do you remove the implant?** |
| --- |
| I never remove hardware |
| < 6 months |
| Between 6 - 12 months |
| Between 12 and 18 months |
| Between 18 - 24 moths |
| > 24 months |

| **4. Who removes implants in your clinic?** |
| --- |
| Yourself |
| Trauma surgeon |
| Every surgeon |
| Resident without supervision |
| Resident with supervision |

| **5. Who taught you how to remove implants?** |
| --- |
| My supervisor |
| Other supervisors |
| Senior resident |
| Junior resident |

| **6. Do you think that operation complications due to implant removal are to be blamed to the level of experience of the surgeon?** |
| --- |
| Yes, I often think so |
| Sometimes, I think so |
| No, I hardly ever think that |

| **7. Do you think that operation complications due to implant removal are to be blamed to the subspecialty of the surgeon?** |
| --- |
| Yes, I often think so |
| Sometimes, I think so |
| No, I hardly ever think that |

| **8. Which problems do you see with some frequency?** |
| --- |
| Nerve damage |
| Bleeding |
| Implant breaks during removal |
| Incorrect instruments present |
| Unplanned fluoroscopy |
| Cold welding |
| Stripping screw head |
| Implant difficult to find |
| Larger incision necessary |
| Operation time longer than planned |
| Implant overgrown by bone |
| never see problems |

| **9. Are there any differences in removing titanium (TAN) plates or nails versus stainless steel (SS)?** |
| --- |
| No experience with titanium (TAN) / stainless steel (SS) |
| No difference TAN / SS |
| TAN easier to remove |
| TAN more difficult to remove |

| **10. Which complication do you see most?** |
| --- |
| Nerve damage |
| Persisting complaints |
| Refracture |
| No complications observed |
| Bleeding |
| Unpleasant scar |
| Wound infection |
| Others |

| **11. Do you think implant removal belongs to trauma surgery?** |
| --- |
| Yes |
| Yes, but practice is different |
| No |
| No opinion |
